# Use of pyridoxine hydrochloride in the interruption of lactation in female dogs with pseudopregnancy

**DOI:** 10.1590/1984-3143-AR2020-0062

**Published:** 2021-03-31

**Authors:** Maíra Corona da Silva, Paula Elisa Brandão Guedes, Fabiana Lessa Silva, Paola Pereira das Neves Snoeck

**Affiliations:** 1 Departamento de Ciências Agrárias e Ambientais, Universidade Estadual de Santa Cruz – UESC, Ilhéus, BA, Brasil

**Keywords:** cabergoline, canine species, pseudociese, vitamin B6

## Abstract

The aim of this research was to evaluate the use of pyridoxine hydrochloride and its associated side effects in the treatment of pseudopregnancy in female dogs. A total of 40 female dogs, with no defined breed, in non-gestational diestrus, with clinical complaint of milk production were selected. The female dogs were divided into four experimental groups of 10 animals each, treated orally for 20 days with 10mg/kg/day (G1) and 50mg/kg/day (G2) of pyridoxine hydrochloride (vitamin B6), 5μg/kg/day of cabergoline (G3), and with a placebo, in the case of the control group (G4). The effects of the treatments on milk production were investigated, as well as possible systemic side effects, macroscopic uterine and ovarian alterations, and uterine histology. During the investigated period, G2 and G3 were equally efficient (P>0.05) in lactation suppression, differing (P>0.05) from the other groups. There were no systemic side effects or uterine changes associated with administration of the studied drug. Vitamin B6 (50mg/kg) has shown to be a safe and economically viable alternative for lactation interruption in female dogs with pseudopregnancy.

## Introduction

Pseudocyesis or pseudopregnancy is a syndrome that affects female dogs in non-gestational diestrus and is characterized by similar physiological changes to those occurring at the end of pregnancy or postpartum. The clinical signs are highly variable, but female dogs generally present mammary development with milk secretion and behavioral alterations, such as restlessness, anorexia, and maternal behavior (Gobello et al., 2001b, d; Feldman and Nelson, 2004; Root et al., 2018). Furthermore, studies indicate that this alteration predisposes the animal to the development of mammary neoplasms (Oliveira et al., 2003). Susceptible female dogs frequently present recurrence in successive estrous (Feldman and Nelson, 2004), developing the syndrome after exposure to progesterone and its subsequent reduction (Gobello et al., 2001b, c, d).

Prolactin (PRL) plays a fundamental role in the pathophysiology of pseudopregnancy in association with abrupt, abnormal progesterone reduction (P4) in the late luteal phase (Concannon, 1989; Gobello et al., 2001b, c, d). Although studies have demonstrated a lack of consistency between the intensity of the clinical signs of pseudocyesis and serum concentrations of PRL (Hoffmann et al., 1992; Harvey et al., 1997; Harvey et al., 1999), the use of ergot derivatives such as metergoline and cabergoline, in pseudopregnancy treatment, highlights the importance of PRL in the etiopathogeny of the syndrome (Gobello et al., 2001c). However, the only drug sold in Brazil for the treatment of canine pseudocyesis is metergoline which, besides having a short half-life, causes intense side effects, such as gastrointestinal manifestations, aggressiveness, vocalization, and hyperexitation (Hamon et al., 1981).

Pyridoxine hydrochloride has also demonstrated as a viable therapeutic alternative for suppressing lactation in women and reducing PRL concentrations (Foukas, 1973; Delitala et al., 1976). Moreover, results of studies on rats treated with vitamin B6 showed a decrease in PRL concentrations, suggesting a dopaminergic effect of pyridoxine on the hypothalamus (Harris et al., 1978). Additionally, a study conducted with French bulldogs found that vitamin B6 (50mg/kg) could interfere in PRL serum concentration in a similar way to cabergoline (5µg/kg) (Silva and Snoeck, 2020), since it acts as a coenzyme in processes of decarboxylation of L-dopa in dopamine, the main inhibiting factor of PRL (Bruice, 2006). Therefore, it is suggested that vitamin B6 may also interfere with the clinical signs of pseudopregnancy, as in the case of ergot-derived drugs (Gobello et al., 2001c). The recommended dose of vitamin B6 for dogs, regardless of development phase, is 1.5 mg/kg of dry food, this being fulfilled by the formulation of commercial dog food (NRC, 2006). An intermediate dose of 50 mg/kg/day, supplied for up to 107 days, proved to be safe and free of undesirable side effects (Phillips et al., 1978).

Thus, in view of the importance of pseudocyesis for animal health and welfare, as highlighted above, and given that the effect of pyridoxine hydrochloride on the lactation of pseudopregnant female dogs has not yet been investigated, the present study sought to evaluate the clinical efficiency of pyridoxine hydrochloride in the treatment of canine pseudocyesis. In addition, the specific objective was also to investigate the occurrence of systemic side effects of administration of the referred drug, as well as possible uterine and ovarian macroscopic changes, and uterine histology modifications.

## Methods

The project was approved by the Ethics Committee of the State University of Santa Cruz (CEUA-UESC) under number 036/2015. Furthermore, the owners of the animals included in this study signed a consent form, agreeing to the dog´s participation.

The study sample was composed of 40 female dogs of any breed, 33 from shelters and seven from private owners. The dogs presented pseudocyesis, were two to six years old and weighed between 5 and 15 Kg. It is worth mentioning that the age of the animals from the shelters was estimated, since it was not possible to obtain exact information in this regard. It was certified that neither elderly animals nor those in recent puberty were included. The criterion for inclusion of the animals in the study was lactic secretion by the mammary gland during the diestrus period, confirmed by vaginal cytology. This criterion was adopted since it is the clinical sign primarily and predominantly reported in pseudopregnant female dogs (Root et al., 2018).

The female dogs were allocated to four experimental groups, each with 10 animals. Group 1 (G1) received pyridoxine hydrochloride at 10mg/kg/day, group 2 (G2) received pyridoxine hydrochloride at 50mg/kg/day, group 3 (G3-positive control) received cabergoline at 5µg/kg/day, and the control group (G4-negative control) received a placebo (empty capsules). The dogs received the drugs in the morning, after feeding, on a daily basis for 20 consecutive days. Administration was made orally, through manipulated capsules delivered according to the weight of each animal. The protocol was initiated for each animal when the beginning of milk production was identified. The animals remained in their homes during the treatment period and were monitored by the owner, who administered the drugs. Regarding the animals in the shelters, the medication was administered by their keepers. Tutors and caregivers were instructed to monitor and report any changes, such as nausea, drooling, vomiting, diarrhea or behavioral changes. The dogs were also clinically re-evaluated for milk production on a weekly basis, through visual inspection and palpation. To this end, a specific researcher visited each dog at the shelter or home to evaluate and record the occurrence of undesirable side effects of the treatment.

PRL levels were monitored on day zero (two hours before the first drug administration) and 120 hours after the start of treatment, in order to verify a possible correlation between serum concentrations of PRL and the intensity of clinical signs of pseudocyesis. To determine PRL levels, blood sampling (5mL) was performed through jugular venipuncture using vacuum tubes with clot activator, with minimal stress to the animals. The vacuum tubes were then centrifuged at 3000 rpm for 7 minutes to obtain serum, divided into aliquots in microcentrifuge tubes (500 µL) and stored at -20° C until laboratory analysis.

It is important to highlight that there was no abandonment of any animals in the study, since all were submitted to ovarian-salpingo-hysterectomy (OSH) at the end of the experimental period (20 days of treatment), by the researcher who accompanied them during the research. The reproductive system was macroscopically analyzed in order to identify the presence of follicular and/or lutein structures in the ovaries and possible pathological changes such as cysts, local inflammation or the presence of content in the uterine lumen. Following evaluation, uterine fragments of 2 cm^2^ were collected through cross-section of the middle third of both uterine horns, which were preserved in 10% formalin until conventional histological processing for inclusion in paraffin and subsequent staining with hematoxylin eosin. The histological findings were characterized according to the previously described methodology (De Bosschere et al., 2001).

The variables were compared through mean, standard deviation, and hypothesis testing. For comparison within the same group, the Wilcoxon test was applied to paired data. The Kruskal-Wallis test was applied for comparisons between the groups before and after each treatment (Milone, 2004; Lehmann and D'abrera, 2006).

## Results

In the first week, the female dogs treated with pyridoxine hydrochloride at a dose of 50mg/kg (G2) and those treated with cabergoline 5µg/kg (G3) showed lactation suppression in 40% (n=4) and 60% (n=6) of the animals investigated, respectively. In the second week, the female dogs treated with pyridoxine hydrochloride at a dose of 10mg/kg (G1), with pyridoxine hydrochloride at 50mg/kg (G2), and with cabergoline 5µg/kg (G3) presented lactation interruption in 20% (n=2), 90% (n=9) and 100% (n=10) of treated animals, respectively. At the end of the third week, 50% of the animals in G1 (n=5), 100% of G2 (n=10), 100% of G3 (n=10), and 20% of G4 (n=2) presented no milk production (Table 1).

**Table 1:** Number and accumulated weekly percentage of pseudopregnant female dogs that manifested lactation suppression with the use of pyridoxine hydrochloride (10mg/kg/day or 50mg/kg/day) or cabergoline (5µg/kg/day).

Group	1^st^ week	2^nd^ week	3^rd^ week
G1 (n=10)	0^B^ (0%)	2^B^ (20%)	5^B^ (50%)	
G2 (n=10)	4^A^ (40%)	9^A^ (90%)	10^A^ (100%)	
G3 (n=10)	6^A^ (60%)	10^A^ (100%)	10^A^ (100%)	
G4 (n=10)	0^B^ (0%)	0^B^ (0%)	2^B^ (20%)	

^A,B^ Different letters in the same column indicate significant difference (P<0.05). G1 (10mg of B6/kg/day), G2 (50mg of B6/kg/day), G3 (5µg of cabergoline/kg/day), and G4 (placebo). n= number of female dogs used/group.

Pyridoxine hydrochloride (50mg/kg) and cabergoline (5µg/kg) were equally efficient (P>0.05) in suppressing lactation and in visual remission of mammary development of treated animals, differing (P<0.05) from females in the other groups in all weeks investigated.

There were no undesirable side effects related to drug administration, such as nausea, vomiting, hyperexcitation, or diarrhea. Furthermore, treatment with vitamin B6 (50 mg/kg/day of pyridoxine hydrochloride) presented lower cost (average cost of treatment of 38.00 Brazilian Reals per animal).

Serum concentrations of PRL in pseudopregnant female dogs were reduced with the administration of pyridoxine hydrochloride at 50mg/kg (G2) and cabergoline at 5µg/kg (G3), these being equally efficient (P>0.05) and differentiated (P<0.05) from the other groups studied (Table 2).

**Table 2:** Averages of PRL serum concentrations in pseudopregnant female dogs at 0h (day of initiation of treatment, before the first administration of drugs) and 120h after administration of cabergoline or pyridoxine hydrochloride.

Treatment	PRL (ng/mL)	
	Day 0	120h
G1	2.9^A^	3.0^A^
G2	1.2^aB^	<0.6^bB^
G3	3.6^aA^	<0.6^bB^
G4	3.1^A^	3.3^A^

^a,b^ Different letters in the same line indicate significant difference (P<0.05). ^AB^Different letters in the same column indicate significant difference (P<0.05). G1 (10mg of B6/kg/day), G2 (50mg of B6/kg/day), G3 (5µg of cabergoline /kg/day), and G4 (placebo).

Eight female dogs from G1 (8/10) and nine from G4 (9/10) had macroscopically identifiable corpus luteum (CL) in at least one of the ovaries at the time of OSH. One G4 (control) animal presented multiple ovarian cysts and uterine lumen with a large volume of purulent content. The other animals did not present macroscopic alterations.

Histological evaluation of the uterine fragments did not reveal alterations that could compromise uterine physiology. A similar morphological pattern was found between the groups (P>0.05), with areas of moderate endometrial hemorrhage in more superficial layers, moderate endometrial hyperemia, and mild endometrial edema, without areas of glandular distension and absence of mononuclear infiltrates. Only one G4 individual had complex cystic endometrial hyperplasia, with cystic dilation of diffuse and accentuated endometrial glands, presence of inflammatory infiltration (macrophages, neutrophils, and plasmocytes), and moderate multifocal myometritis.

## Discussion

The similar results found between the groups that received pyridoxine hydrochloride (50mg/kg) and cabergoline (5µg/kg) showed the importance of PRL in the development and maintenance of pseudopregnancy (Okkens et al., 1997). In addition, these results indicate that pyridoxine hydrochloride has similar effects to the commercial drug currently used in veterinary clinical routine for suppression of pseudopregnancy in female dogs. The improvement of clinical signs in the female dogs of the other groups may be associated with the known tendency of gradual remission of pseudocyesis (Gobello et al., 2001b, d).

The absence of undesirable side effects related to the administration of vitamin B6 and its lower cost demonstrated that its use may be an efficient and affordable option for the treatment of lactation in pseudopregnant female dogs, since the protocol of treatment with cabergoline (5µg/kg/day) had an average cost of 80.00 Brazilian Reals per animal, 42 Reals more than treatment with pyridoxine.

The efficiency in reducing PRL serum concentrations of pseudopregnant female dogs, both with pyridoxine hydrochloride 50mg/kg (G2) and with cabergoline 5µg/kg (G3), confirm the inhibitory action of vitamin B6 on PRL serum concentration in female dogs. This may be justified by the fact that pyridoxine hydrochloride, as well as cabergoline, acts as a dopaminergic agonist, favoring the synthesis of dopamine, which, in turn, acts as a prolactin inhibitor (Delitala et al., 1976; Harris et al., 1978; Bruice, 2006).

G2 female dogs had a lower PRL serum concentration than those in other groups (P<0.05) before treatment, but in the clinical evaluation these animals could not be distinguished from the other groups regarding signs of pseudocyesis. It is possible that clinical manifestations are not only associated with PRL concentration, but also with the bioactivity of the molecule and its affinity for receptors, since the canine species has different molecular forms of PRL with similar serum concentrations, denominated as large (>67kDa), native (23kDa), and fragmented (<20kDa) (Gobello et al., 2001a). In humans, the monomeric form of PRL (23kDa) presents higher serum concentration (80-90%) in normal individuals, with high affinity to receptors and high bioactivity. On the other hand, macroprolactins (48-56kDa) and glycosylated PRLs (25kDa) are less immunoreactive and present less biological activity (Sinha, 1995; Hattori and Inagaki, 1997; Vieira, 2002). It is possible that the heterogeneity of the PRL molecule in canine species also influences clinical findings, as occurs in humans. However, the bioactivity of each molecule in dogs remains unknown (Gobello et al., 2001a).

Macroscopic identification of the corpus luteum (CL) in at least one of the ovaries of the G1 and G4 female dogs, at the time of OSH, demonstrates the importance of PRL in maintaining the CL. The luteotropic effect of PRL in dogs ensures that a higher concentration of progesterone is maintained regardless of whether the dogs are pregnant or not and is essential for the preparation and maintenance of lactation (Onclin and Verstegen, 1997; Kowalewski et al., 2011; Kowalewski, 2014; Rufo et al., 2016). Thus, it is possible that the reduction in PRL concentrations in dogs through the use of pyridoxine (50mg/kg/day) or cabergoline (5µg/kg/day) has stimulated premature luteal regression. Therefore, other studies should be conducted to verify the action of pyridoxine hydrochloride on progesterone secretion and abortion induction.

Regarding the microscopic changes observed in the analyzed samples, these may be associated with estrogenic influence of the estrous cycle. In relation to the dog belonging to G4 that presented complex cystic endometrial hyperplasia-pyometra, this pathology was considered pre-existing and associated with the presence of multiple ovarian cysts identified in the macroscopic evaluation.

## Conclusion

Vitamin B6 (50mg/kg) proved to be a safe and economically viable alternative for the reduction of PRL serum concentrations, remission of mammary development, and interruption of lactation in pseudpregnant female dogs, presenting satisfactory results with 14 days of treatment. These results indicate an affordable alternative available to owners, free from undesirable side effects for treated animals and with similar therapeutic effects to the commercial drug currently used in veterinary clinical routine.
